# Disparities in appendicitis rupture rate among mentally ill patients

**DOI:** 10.1186/1471-2458-7-331

**Published:** 2007-11-15

**Authors:** Jen-Huoy Tsay, Cheng-Hua Lee, Yea-Jen Hsu, Pen-Jen Wang, Ya-Mei Bai, Yiing-Jenq Chou, Nicole Huang

**Affiliations:** 1 Department of Social Work, College of Social Science, National Taiwan University, Taipei, Taiwan, R.O.C; 2 Institute of Hospital and Health Care Administration, School of Medicine, National Yang Ming University, Taipei, Taiwan, R.O.C; 3 Bureau of National Health Insurance, Taipei, Taiwan, R.O.C; 4 Department of Health Policy and Management, Johns Hopkins Bloomberg School of Public Health, Baltimore, Maryland, USA; 5 Psychiatry Department, Taipei Veterans General Hospital, Taipei, Taiwan, R.O.C; 6 Institute of Public Health, School of Medicine, National Yang Ming University, 155 Li-Nong Street, Section 2, Taipei 112, Taiwan, R.O.C

## Abstract

**Background:**

Many studies have been carried out that focus on mental patients' access to care for their mental illness, but very few pay attention on these same patients' access to care for their physical diseases. Acute appendicitis is a common surgical emergency. Our population-based study was to test for any possible association between mental illness and perforated appendicitis. We hypothesized that there are significant disparities in access to timely surgical care between appendicitis patients with and without mental illness, and more specifically, between patients with schizophrenia and those with another major mental illness.

**Methods:**

Using the National Health Insurance (NHI) hospital-discharge data, we compared the likelihood of perforated appendix among 97,589 adults aged 15 and over who were hospitalized for acute appendicitis in Taiwan between the years 1997 to 2001. Among all the patients admitted for appendicitis, the outcome measure was the odds of appendiceal rupture vs. appendicitis that did not result in a ruptured appendix.

**Results:**

After adjusting for age, gender, ethnicity, socioeconomic status (SES) and hospital characteristics, the presence of schizophrenia was associated with a 2.83 times higher risk of having a ruptured appendix (odds ratio [OR], 2.83; 95% confidence interval [CI], 2.20–3.64). However, the presence of affective psychoses (OR, 1.15; 95% CI: 0.77–1.73) or other mental disorders (OR, 1.58; 95% CI: 0.89–2.81) was not a significant predictor for a ruptured appendix.

**Conclusion:**

These findings suggest that given the fact that the NHI program reduces financial barriers to care for mentally ill patients, they are still at a disadvantage for obtaining timely treatment for their physical diseases. Of patients with a major mental illness, schizophrenic patients may be the most vulnerable ones for obtaining timely surgical care.

## Background

Acute appendicitis is a common surgical emergency. A growing inventory of medical literature has used differential rates of ruptured appendix as an indicator of a potential barrier to the accessibility of medical care [1-7]. Although symptomatic acute appendicitis is not preventable, timely treatment of symptomatic acute appendicitis can help to prevent appendiceal perforation, which is associated with severe morbidity and elevated mortality[1,8-10]. According to previous literature, the strongest predictor of perforation is postponed surgery in excess of 12 to 24 hours after the symptoms of appendicitis first occured. Previous reports observed higher rates of appendiceal perforation among people without insurance, minorities, young children, the elderly and men [1-3,7,8,11,12]. However, none of these studies had systematically examined whether there was a difference between patients, with or without a mental disorder. In addition to direct physiological mechanisms, hindered access to necessary medical care may lead to a greater number of adverse outcomes of co-morbidity among patients suffering from mental illness [13-20].

Patients with a major mental disorder, such as schizophrenia or affective psychosis, may be at a higher risk of encountering hindered access to care for their physical diseases, possibly due to their impaired capacity to recognize or articulate the emerging medical illness [15,18,19,21,22]. Therefore, not only does the access of mentally ill patients to mental health services deserves policy attention, but their access to care for their physical diseases should also not be overlooked. For example, the typical consequences of schizophrenia, such as cognitive impairment, social isolation, suspicion and possible pain insensitivity may cause mentally-ill patients to be less likely to seek medical help, promptly report physical symptoms or follow prescribed treatment [23-29]. Also, physicians' lack of experience with mentally ill patients may lead to misdiagnosis or delayed diagnoses in certain cases. Some psychotic symptom such as somatic delusions may lead physicians whom are less experienced in treating mentally ill patients to overlook some of the physical symptoms presented. Furthermore, many psychiatric institutions may not have sufficient diagnostic equipment or surgical facilities to provide timely care for the physical ailments of their patients. Factors related to patient, physician and institution, may impede mentally ill patients, particularly schizophrenic patients, from receiving timely care [14,15,20,22,30].

Although only anecdotal evidence and a few reports based on small samples suggest that schizophrenia patients may have a higher rate of perforated appendicitis [31], there is no large-scale study available in the medical literature on this topic. Moreover, in addition to providing insurance [32,33], a large variety of extensive efforts are devoted to assure access to mental health services by mentally ill patients by addressing cultural, linguistic, and other barriers. However, we should not limit our attention to these patients' access to care for mental diseases, but also their access to care for physical diseases. The implementation of the National Health Insurance (NHI) in Taiwan in 1995 successfully reduced financial barriers to access care for all including the mentally ill. Nevertheless, the purpose of this study was to investigate whether disparities in access to care for appendicitis still exist between patients with and without mental illness, and more specifically, between patients with schizophrenia and other major mental illness. Our work contributes to the existing literature by conducting a first population-based study to systematically examine the association between schizophrenia, affective psychosis, other major mental illnesses, and appendiceal perforation. Although our findings may not lead to important hints about the etiology or pathophysiology of mental illnesses, they can help to identify disparities so that we can devise strategies to reduce these disparities in access to timely surgical care for physical illnesses between patients with and without mental illness, and between patients with different mental illnesses.

## Methods

### Data and study sample

NHI claims data were used to identify all discharges in Taiwan. The NHI enrollment files, which record the basic personal information of the NHI beneficiaries for insurance purpose, were used to provide information on patients' insurable wage and enrollment category. After excluding those with incomplete enrollment information (2.4%) and patients with foreign nationality (2.9%), the sample consisted of all the 97,589 hospitalizations of Taiwan residents aged 15 and over for the period of 1997 to 2001, with a principal diagnosis of acute appendicitis, according to the International Classification of Diseases, Ninth Revision, Clinical Modification (ICD-9-CM; codes 540.0, 540.1, or 540.9). Because of possible unfamiliarity and errors with data collection and reporting practices in the first two years of the NHI program (1995–1996), we chose to disregard the data of the first two years (1995–1996) from the NHI databases in order to assure better data quality. Only the data of 1997 and the following years were used for analyses. Furthermore, due to limitations in data availability, we could only obtain data up to the year 2001. Children under the age of 15 years were excluded because patterns of mental disease and appendicitis may differ between children and adults. Also, unlike adults, children rely heavily on their parents or other adults for access and use of medical services. Anonymous personal identification numbers and dates of birth were used to link the NHI claims data and the enrollment file. In order to protect privacy and assure confidentiality, all unique personal identifiers were encrypted by the Bureau of National Health insurance and any information which has the potential to identify the study subject was deleted before being released to the researchers. The confidentiality assurances were addressed by abiding the data regulations of the Bureau of National Health Insurance.

### Variables

The outcome measurement was appendiceal rupture (ICD_9_CM codes 540.0 and 540.1) in all patients hospitalized for acute appendicitis. Appendicitis, not resulting in a ruptured appendix, was defined as ICD_9_CM code 540.9. Independent variables included mental disorders, personal characteristics (age, sex, ethnicity and socioeconomic status (SES)) and hospital characteristics (ownership, accreditation level, patient volume and geographic location).

#### Mental disorder status

The Major Diseases Database, again managed by the Bureau of National Health Insurance, was used to identify individuals with major medical diseases. The Injury Severity Score (ISS) is a widely recognized and anatomically based injury classification scheme [34]. ISS was first introduced in 1971 by a joint committee in the United States. It is commonly used to assess the overall severity of a patient. The Bureau adopts ISS to identify an official list of severe diseases including major mental disorders such as schizophrenia and affective psychosis [35]. In Taiwan, people with any of the listed major mental or non-mental diseases, who have confirmed diagnoses from psychiatrists and/or physicians, can apply for a "major disease/injury card." Cardholders are exempted from the cost-sharing required under the NHI program. In this study sample, 435 patients (0.4%) were cardholders with major mental diseases. Schizophrenia (259 patients) and affective psychoses (123 patients) accounted for more than 80% of these appendicitis patients with major mental diseases (see Table 1), while there were only 53 mentally ill patients suffering from major mental disorders other than schizophrenia or affective psychoses. To further categorize these remaining non-schizophrenic and non-affective psychoses patients into more detailed categories would result in insufficient sample sizes for statistical analyses. Hence, we divided those patients with and without major mental co-morbidities into 4 categories: no major mental disorder, schizophrenia (ICD_9_CM code 295.xx), affective disorders (ICD_9_CM code 296.xx), and other major mental disorders (ICD_9_CM codes 290.xx-294.xx, 297.xx-319.xx). This resulted in a total of four categories, to be used as major independent variables. Previous researches show that the Major Diseases Database offers reliable information on the identification of a representative sample of patients with severe psychiatric disorders among the NHI enrollees in Taiwan [36-38].

**Table 1:** Characteristics of appendicitis patients aged 15 or above and their admitting hospitals in Taiwan, 1997–2001

	Non-Ruptured (n = 73,015)		Ruptured (n = 24,574)		Total (n = 97,589)
			
Characteristics	n	(%)	n	(%)	n
Mental disorder status					
No major mental disorder	72,758	(74.9)	24,396	(25.1)	97,154
Schizophrenia	138	(53.3)	121	(46.7)	259
Affective psychosis	89	(72.4)	34	(27.6)	123
Other major mental disorders	30	(56.6)	23	(43.4)	53
Gender					
Female	36,416	(78.4)	10,024	(21.6)	46,440
Male	36,599	(71.6)	14,550	(28.5)	51,149
Age (years)					
15–24	23,178	(81.4)	5,281	(18.6)	28,459
25–34	18,341	(81.2)	4,261	(18.9)	22,602
35–44	14,518	(75.9)	4,619	(24.1)	19,137
45–54	7,929	(69.7)	3,440	(30.3)	11,369
55–64	4,247	(62.1)	2,597	(38.0)	6,844
65–74	3,277	(55.1)	2,669	(44.9)	5,946
≥ 75	1,525	(47.2)	1,707	(52.8)	3,232
Ethnicity					
Non-aboriginal	70,535	(74.8)	23,770	(25.2)	94,305
Aboriginal	2,480	(75.5)	804	(24.5)	3,284
Socioeconomic status					
≥ NT$40,000	11,211	(75.3)	3,685	(24.7)	14,896
NT$20,000 ~ NT$39,999	17,244	(77.2)	5,103	(22.8)	22,347
<NT$20,000	21,846	(77.0)	6,523	(23.0)	28,369
Without a well-defined monthly wage	22,714	(71.0)	9,263	(29.0)	31,977
Hospital ownership					
Public	15,529	(76.7)	4,719	(23.3)	20,248
Nonprofit	33,578	(74.4)	11,544	(25.6)	45,122
Profit	23,908	(74.2)	8,311	(25.8)	32,219
Hospital accreditation level					
District Hospital-nonteaching	14,093	(73.5)	5,072	(26.5)	19,165
District Hospital-teaching	13,331	(80.5)	3,225	(19.5)	16,556
Regional Hospital	28,810	(75.1)	9,565	(24.9)	38,375
Medical Center	16,781	(71.4)	6,712	(28.6)	23,493
Hospital volume					
≥ 360	18,370	(72.8)	6,851	(27.2)	25,221
210–360	19,120	(74.5)	6,559	(25.5)	25,679
80–210	17,907	(77.1)	5,324	(22.9)	23,231
0–80	17,618	(75.1)	5,840	(24.9)	23,458
Hospital's geographic location					
Taipei Branch	23,536	(77.0)	7,040	(23.0)	30,576
Northern Branch	12,999	(77.7)	3,739	(22.3)	16,738
Central Branch	11,790	(70.6)	4,907	(29.4)	16,697
Southern Branch	9,789	(72.7)	3,676	(27.3)	13,465
Kao-Ping Branch	12,693	(73.6)	4,553	(26.4)	17,246
Eastern Branch	2,208	(77.0)	659	(23.0)	2,867
Year					
1997	13,649	(72.7)	5,129	(27.3)	18,778
1998	13,892	(73.8)	4,939	(26.2)	18,831
1999	14,435	(74.2)	5,017	(25.8)	19,452
2000	15,146	(76.1)	4,757	(23.9)	19,903
2001	15,893	(77.1)	4,732	(22.9)	20,625

#### Patient characteristics

The NHI claims data provides information on each patient's unique, and anonymous, personal identification number, date of birth, gender, diagnoses and unique hospital identifier. Based on the previous findings [1,7-9], we included age and gender as possible confounders. Family registration files helped identify the ethnicity of individual patients. We categorized patients into 2 groups: aboriginal and non-aboriginal. According to previous literature, the aboriginal population may have a higher risk of appendiceal rupture than non-aboriginal [1,12,39]. The NHI Enrollment files were used to provide SES information about the study subjects. Enrollment in the NHI program is mainly through employers; the NHI program offers universal and comprehensive coverage financed by payroll deduction for those people with a well-defined monthly wage and by a head tax for those without a well-defined monthly wage. Those people without a well-defined monthly wage tend to be vulnerable subpopulations such as farmers and low income people and hence, were grouped under one category. People with well-defined monthly wages were classified into three categories: ≥ NT$40,000; NT$20,000 ~ NT$39,999; and <NT$20,000. Thus, the SES variable had four categories in total. Dependents of the insured were classified under the same four categories. Sensitivity analyses were conducted for the different SES grouping schemes and the results remained robust. Although the NHI program is expected to reduce financial barriers to health services covered by the NHI program, people still need to pay costs such as transportation and taking leave from work. So this study included SES as a possibleconfounder.

#### Hospital characteristics

As hospital characteristics such as hospital ownership may have an influence on a physician's diagnostic and reporting practices, the study included hospital characteristics as control variables. The NHI hospital registry provides information on ownership, accreditation level and geographic location of the hospitals providing treatment. Hospital ownership was categorized into public, private nonprofit and private for profit hospitals. Under the current accreditation system in Taiwan, all medical institutions are classified into four major accreditation levels: academic medical center, regional hospital, local community teaching hospital and local community non-teaching hospital. The accreditation criteria include infrastructure, capacity, manpower, volume, management and administrative processes. The geographic area of the hospitals was determined by the 6 regional branches of the Bureau of National Health Insurance, located in the Taipei, Northern, Central, Southern, Kao-Ping and Eastern regions of Taiwan. According to small area variation literature, providers in different regions may vary in their practice patterns. And, as these regions may vary in their availability of either medical providers or facilities for surgical care and mental services, we included this variable as a confounder [11]. The geographic location served as a proxy for availability of medical resources and physical accessibility to an appropriate medical provider. Patient volume was defined as the annual number of appendicitis patients treated by each hospital [1].

For statistical analyses, a multiple logistic regression model was constructed to estimate adjusted odds ratios. Confounding factors, which were adjusted for, included patient characteristics (age, sex, ethnicity, SES), hospital characteristics (ownership, accreditation level, volume, geographic location) and the year dummies. After controlling for these confounders, the adjusted rates of ruptured appendix by mental disorder status were also determined. Alternative models controlling physical co-morbidities such as diabetes and neoplasm were carried out and the results remained robust. All analyses were conducted using SAS 8.2 and STATA 8.0 statistical software packages.

## Results

Table 1 describes the characteristics of the study sample according to the presence or absence of a ruptured appendix. A ruptured appendix occurred in 46.7 percent of the schizophrenic patients; in 43.4 percent of the patients with other major mental disorders; in 27.6 percent of the patients with affective psychoses; and in 25.1 percent of the patients with no major mental diseases. More ruptured cases were found among males and older patients. The rate of ruptured appendix decreased from 27.3% in 1997 to 22.9% in 2001. Overall, although the total number of symptomatic acute appendicitis increased from 18,778 to 20,625, the incidence rate remained relatively constant (approximately 0.1%) due to the natural population increase and the rising NHI enrollment rate during the study period.

Table 2 presents the results of simple and multiple logistics regression analyses. In the simple logistic regression analysis, schizophrenic patients (OR: 2.61, 95% CI: 2.05–3.34) and other mental disorder patients (OR: 2.29, 95% CI: 1.33–3.94) with appendicitis were significantly more likely than the reference group (patients with appendicitis, but without any major mental diseases) to experience appendiceal rupture. In contrast to schizophrenic patients and patients with other major mental disorders, patients with affective psychoses (OR: 1.14, 95% CI: 0.77–1.69) were not significantly more likely to have appendiceal rupture than the reference group. Other significant patient factors found using simple logistic regression analysis were age, gender, and SES.

**Table 2:** Results of simple and multiple logistic regression for appendiceal perforation, appendicitis patients in Taiwan (aged 15 or above), 1997–2001

Characteristics	Unadjusted OR	95% CI	Adjusted OR	95% CI
Mental disorder status				
No major mental disorder	1.00		1.00	
Schizophrenia	2.61 ***	2.05–3.34	2.83 ***	2.20–3.64
Affective psychosis	1.14	0.77–1.69	1.15	0.77–1.73
Other major mental disorders	2.29 **	1.33–3.94	1.58	0.89–2.81
Gender				
Female	1.00		1.00	
Male	1.44 ***	1.40–1.49	1.46 ***	1.42–1.51
Age (years)				
15–24	1.00		1.00	
25–34	1.02	0.98–1.07	1.00	0.96–1.05
35–44	1.40 ***	1.34–1.46	1.37 ***	1.31–1.43
45–54	1.90 ***	1.81–2.00	1.85 ***	1.76–1.95
55–64	2.68 ***	2.53–2.84	2.54 ***	2.40–2.70
65–74	3.57 ***	3.37–3.79	3.27 ***	3.08–3.49
≥ 75	4.91 ***	4.56–5.30	4.60 ***	4.25–4.98
Ethnicity				
Non-aboriginal	1.00		1.00	
Aboriginal	0.96	0.89–1.04	1.14 **	1.04–1.24
SES				
≥ NT$40,000	1.00		1.00	
NT$20,000 ~ NT$39,999	0.90 ***	0.86–0.95	1.03	0.98–1.08
<NT$20,000	0.91 ***	0.87–0.95	1.04	0.99–1.10
Without a well-defined monthly wage	1.24 ***	1.19–1.30	1.09 ***	1.04–1.14
Hospital ownership				
Public	1.00		1.00	
Nonprofit	1.13 ***	1.09–1.18	1.19 ***	1.14–1.25
Profit	1.14 ***	1.10–1.19	1.38 ***	1.30–1.46
Hospital accreditation level				
District Hospital-nonteaching	1.00		1.00	
District Hospital-teaching	0.67 ***	0.64–0.71	0.75 ***	0.70–0.79
Regional Hospital	0.92 ***	0.89–0.96	1.11 **	1.03–1.19
Medical Center	1.11 ***	1.06–1.16	1.38 ***	1.27–1.51
Hospital volume				
≥ 360	1.00		1.00	
210–360	0.92 ***	0.88–0.96	1.00	0.96–1.05
80–210	0.80 ***	0.76–0.83	1.04	0.98–1.10
0–80	0.89 ***	0.85–0.93	1.11 **	1.03–1.20
Hospital's geographic location				
Taipei Branch	1.00		1.00	
Northern Branch	0.96	0.92–1.01	1.00	0.95–1.05
Central Branch	1.39 ***	1.33–1.45	1.33 ***	1.27–1.39
Southern Branch	1.26 ***	1.20–1.31	1.25 ***	1.19–1.32
Kao-Ping Branch	1.20 ***	1.15–1.25	1.14 ***	1.09–1.20
Eastern Branch	1.00	0.91–1.09	0.91	0.82–1.01
Year				
1997	1.00		1.00	
1998	0.95 *	0.90–0.99	0.96	0.92–1.01
1999	0.92 **	0.88–0.97	0.93 **	0.88–0.97
2000	0.84 ***	0.80–0.87	0.81 ***	0.77–0.85
2001	0.79 ***	0.76–0.83	0.77 ***	0.74–0.81

*** p < 0.001, ** p < 0.01, * p < 0.05

After adjusting for age, gender, ethnicity, SES, and hospital characteristics, the odds ratio increased from 2.61 to 2.83 and schizophrenic patients were still the most likely among all of the patients with appendicitis to experience appendiceal rupture. The next most likely were patients with other major mental disorders, but the risk was no longer significant (OR: 1.58, 95% CI: 0.89–2.81) after taking patient characteristics, hospital characteristics, and years into consideration. One plausible explanation may be that as age is strongly associated with rupture according to previous research, age might be a most likely factor to reduce the odds ratio for the "other" group in the multivariate analysis. Also, after taking more variables into consideration, it might lose degrees of freedom in the multivariate model and hence the odds ratio might become statistically insignificant. Similar to the results of simple logistic analysis, after adjustment, patients with affective psychoses (OR: 1.15, 95% CI: 0.77–1.73) remained insignificantly associated with elevated ruptured appendix rates.

As for the other characteristics, the multivariate results were consistent with previous findings [1,7-11,40]. For adults, increasing age, being male, and lower SES were also significantly associated with a higher risk of perforation. In addition, after adjusting for other patient and hospital characteristics, aboriginals were at significantly higher risk of having appendiceal perforation than non-aboriginals.

Table 3 presents adjusted perforation rates by mental disorder status, patient and hospital characteristics. Of all patients with symptomatic acute appendicitis, schizophrenic patients had the highest perforation rate (46.9%) whereas the patients with no major mental disorders had the lowest perforation rate (23.8%). Patients with affective psychoses and those with other major mental disorders had rates of 26.5% and 33.1%, respectively.

**Table 3:** Adjusted perforation rates by patient and hospital characteristics, appendicitis patients in Taiwan (aged 15 or above), 1997–2001

Characteristics	Adjusted ruptured rate (%)	95% CI
Mental disorder status		
No major mental disorder	23.8	23.5–24.1
Schizophrenia	46.9	40.8–53.2
Affective psychosis	26.5	19.3–35.1
Other major mental disorders	33.1	21.8–46.8
Gender		
Female	20.4	20.1–20.8
Male	27.3	26.9–27.7
Age (years)		
15–24	18.5	18.1–19.0
25–34	18.5	18.0–19.0
35–44	23.7	23.1–24.3
45–54	29.6	28.8–30.5
55–64	36.6	35.5–37.8
65–74	42.7	41.3–44.0
≥ 75	51.1	49.3–52.9
Ethnicity		
Non-aboriginal	23.8	23.5–24.1
Aboriginal	26.2	24.6–27.9
SES		
≥ NT$40,000	23.0	22.3–23.7
NT$20,000 ~ NT$39,999	23.5	23.0–24.1
<NT$20,000	23.8	23.3–24.3
Without a well-defined monthly wage	24.6	24.1–25.1
Hospital ownership		
Public	20.6	20.0–21.3
Nonprofit	23.7	23.2–24.1
Profit	26.4	25.7–27.0
Hospital accreditation level		
District Hospital-nonteaching	22.6	21.7–23.6
District Hospital-teaching	17.9	17.3–18.6
Regional Hospital	24.5	24.0–25.0
Medical Center	28.8	27.9–29.7
Hospital volume		
≥ 360	23.2	22.5–24.0
210–360	23.3	22.7–23.9
80–210	23.9	23.3–24.5
0–80	25.2	24.3–26.0
Hospital's geographic location		
Taipei Branch	22.1	21.6–22.6
Northern Branch	22.1	21.4–22.8
Central Branch	27.4	26.6–28.1
Southern Branch	26.2	25.4–27.0
Kao-Ping Branch	24.4	23.8–25.1
Eastern Branch	20.5	19.0–22.2
Year		
1997	26.1	25.5–26.8
1998	25.4	24.7–26.0
1999	24.7	24.1–25.3
2000	22.2	21.6–22.8
2001	21.5	20.9–22.1

## Discussion

This population-based study found that for all patients with mental illness, the strongest positive association between mental illness and ruptured appendix is for patients with schizophrenia, independent of other risk factors found in previous literature, such as age, sex, SES, ethnicity and hospital characteristics. Previous research indicates that, on average, schizophrenic patients have a 2 to 4 times higher mortality rate than the general population, although this excess mortality has been mainly associated with an elevated risk for suicides or traumatic injuries [23,41-43]. One extreme example is from a paper by Munk-Jorgensen (2000), which indicated that although some other factors could also have contributed to the findings, the rate ratio for schizophrenic patients' admissions to somatic hospitals for acute and potentially lethal diseases is greater than 1, up to a maximum of 4.15 for severe heart failure [17]. Our finding of OR = 2.83 for ruptured appendix among schizophrenic individuals is consistent with previous reports of excess adverse outcomes among schizophrenic patients. Excess adverse outcomes from physical causes among patients with schizophrenia are a serious problem.

Furthermore, not all mentally ill patients are equally vulnerable. Compared to patients with other mental illness, a much higher rate of ruptured appendix was identified among schizophrenic patients in this study. This finding suggests a substantial gap in accessibility to necessary medical care, between patients with and without schizophrenia. The schizophrenic patient's possible insensitivity to pain, cognitive or social impairment, general or surgical practitioner's inexperience in treating schizophrenic patients, inadequate diagnostic or surgical facilities in psychiatric institutions, poor family support, other health system or psychosocial factors which may lead to misdiagnosis, delayed diagnosis and delay in appropriate treatments, could partially explain the excessively higher rates of ruptured appendix observed among schizophrenic patients compared to patients without a mental illness. In addition, some reported pain insensitivity in schizophrenic patients may make them particularly vulnerable to adverse consequences of physical diseases that are typically pain-causing [25-27]. Although some pain insensitivity in patients with schizophrenia has been reported in the literature and the phenomenon has been explained by several advanced theories [26,28], some previous studies suggest that it is not well recognized or understood in general or surgical practice [26,29,44].

The large sample size and the robust adjustment for comprehensive patient and hospital characteristics have allowed this study to obtain a more precise estimate of the independent contribution of schizophrenia to the rates of perforated appendix. Controlling for hospital characteristics was done so as to reconcile a possible variation among physicians in diagnostic and reporting practices. Furthermore, we studied admissions for acute appendicitis only, rather than for appendectomy, in order to avoid including admissions for reasons other than acute appendicitis.

Several study limitations should be noted. First, misdiagnosis of acute appendicitis may lead to an underestimation of perforation rate. Misdiagnosis could be differential or non-differential between schizophrenic patients and others. According to the previous literature [45-47], the misdiagnosis rate of acute appendicitis ranges from 15% to 40%. The negative association between schizophrenia and acute appendicitis, and the under-reporting of medical illnesses in schizophrenic patients found in previous studies imply that overdiagnosis of acute appendicitis may be more prevalent in non-schizophrenic patients than in schizophrenic patients [23,48-50]. Assuming an extreme differential case, where the reference patient group (who did not have any major diseases) had a 40% overdiagnosis rate and the schizophrenic patients had no misdiagnosis, our estimate of the odds ratio between schizophrenic patients and reference group (patients with no major diseases) would be an overestimation. On the other hand, if the misdiagnosis was non-differential, the odds ratio would be larger than the one observed in this study. Due to the lack of pathology, outpatient and emergency room data, we were unable to address the possible misdiagnosis issue in this study. Future research with more comprehensive data may help in this regard. Second, our reliance on the NHI major disease files for mental disorder case definitions may be a problem. The patients with a major disease/injury card of a listed major mental diseases, must have a confirmed diagnosis from a psychiatrist that long-term treatment is necessary. More than six months psychiatric treatment duration is required to validate the necessity of long-term treatment. Hence, because of these stringent criteria, the major disease file is likely to capture those patients with a chronic severe mental disorder. However, it may not have captured all patients with schizophrenia. Furthermore, the stigma associated with schizophrenia may discourage some schizophrenic patients from applying for a major disease card, so these patients may also not be captured by the major disease file. In addition, misclassification of patients with schizophrenia into other groups may also lead to an underestimation of the differences between patients with and without schizophrenia. Moreover, the sample size limitation restricts us to conducting more detailed interaction analyses such as the interaction between mental disorder and age. An insufficient sample size may result in the large standard error. Future research with a larger sample size may help to detect differential mental disorder impacts on ruptured appendicitis between different age groups. Finally, our proxies of SES were limited to individual-level insurable wages in the enrollment category and so they may not be comprehensive.

## Conclusion

The findings of our study suggest that mentally ill patients are at a disadvantage for obtaining timely treatment for their physical diseases. Of patients with a major mental illness, schizophrenic patients may be the most vulnerable ones due to biological, health care system and psychosocial factors as discussed above. Atypical presentations in schizophrenic patients raise serious clinical concern for an increased risk of misdiagnosis regarding evaluation as well as delayed treatment. How to reduce disparities in physical health for the mentally ill patients is a complex issue and will need a multidimensional strategy to address the possible barriers among this vulnerable subpopulation. For example, a better integrated care delivery system such as case management programs for a closer integration of physical and mental health services of mentally ill patients would be of some benefit. It would allow physicians to have a better understanding of a patient's history and present status, which could help physicians distinguish between symptoms of serious illnesses and noise. In addition, a higher perforating rate among mentally-ill patients may also be related to patient factors and system factors. Therefore, educational interventions such as providing more health information or training to patients' families or caregivers may help to reduce delayed presentation for care due to the pain insensitivity or delayed personal recognition of the potentially-serious nature of symptoms. Organizational strategies may also help to facilitate or reduce possible delays in diagnosis or treatment processes. For mentally ill patients, not only are their mental health concerns important, their physical health issues are equally critical. Taiwan's experience suggests that improving health for mentally ill patients is more than a matter of finances. More attention is required on how some health care features are organized, as well as educational interventions. Both may help to reduce possible non-financial barriers and reduce the excess morbidity and complications for this vulnerable population, both in Asia and around the world.

## Competing interests

The author(s) declare that they have no competing interests.

## Authors' contributions

JHT and NH planned the study, led the writing and supervised all aspects of its implementation. CHL and YJC synthesized analyses and contributed to the writing of the article. YJH, PJW and YMB contributed to the design, assisted in statistical analyses, and commented on the interpretation of the results. All authors helped to conceptualize ideas, interpret findings, and review drafts of the manuscript. All authors read and approved the final manuscript.

## Pre-publication history

The pre-publication history for this paper can be accessed here:


